# Mapping Astrocyte Transcriptional Signatures in Response to Neuroactive Compounds

**DOI:** 10.3390/ijms22083975

**Published:** 2021-04-12

**Authors:** Debosmita Sardar, Brittney Lozzi, Junsung Woo, Teng-Wei Huang, Caroline Cvetkovic, Chad J. Creighton, Robert Krencik, Benjamin Deneen

**Affiliations:** 1 Center for Cell and Gene Therapy, Stem Cells and Regenerative Medicine Center, Baylor College of Medicine, Houston, TX 77030, USA; Debosmita.Sardar@bcm.edu (D.S.); Junsung.Woo@bcm.edu (J.W.); thuang@bcm.edu (T.-W.H.); 2 Genetics and Genomics Graduate Program, Baylor College of Medicine, Houston, TX 77030, USA; Brittney.Lozzi@bcm.edu; 3 Department of Neurosurgery, Center for Neuroregeneration, Houston Methodist Research Institute, Houston, TX 77030, USA; ccvetkovic@houstonmethodist.org (C.C.); rkrencik@houstonmethodist.org (R.K.); 4 Division of Biostatistics, Dan L. Duncan Cancer Center, Baylor College of Medicine, Houston, TX 77030, USA; creighto@bcm.edu; 5 Department of Medicine, Baylor College of Medicine, Houston, TX 77030, USA; 6 Department of Neuroscience, Baylor College of Medicine, Houston, TX 77030, USA; 7 Department of Neurosurgery, Baylor College of Medicine, Houston, TX 77030, USA

**Keywords:** astrocyte, neuron, noradrenaline, transcriptomic, chromatin

## Abstract

Astrocytes play central roles in normal brain function and are critical components of synaptic networks that oversee behavioral outputs. Despite their close affiliation with neurons, how neuronal-derived signals influence astrocyte function at the gene expression level remains poorly characterized, largely due to difficulties associated with dissecting neuron- versus astrocyte-specific effects. Here, we use an in vitro system of stem cell-derived astrocytes to identify gene expression profiles in astrocytes that are influenced by neurons and regulate astrocyte development. Furthermore, we show that neurotransmitters and neuromodulators induce distinct transcriptomic and chromatin accessibility changes in astrocytes that are unique to each of these neuroactive compounds. These findings are highlighted by the observation that noradrenaline has a more profound effect on transcriptional profiles of astrocytes compared to glutamate, gamma-aminobutyric acid (GABA), acetylcholine, and serotonin. This is demonstrated through enhanced noradrenaline-induced transcriptomic and chromatin accessibility changes in vitro and through enhanced calcium signaling in vivo. Taken together, our study reveals distinct transcriptomic and chromatin architecture signatures in astrocytes in response to neuronal-derived neuroactive compounds. Since astrocyte function is affected in all neurological disorders, this study provides a new entry point for exploring genetic mechanisms of astrocyte–neuron communication that may be dysregulated in disease.

## 1. Introduction

Astrocytes are a non-neuronal cell type that comprise at least 30% of the cellular constituency of our brains [1]. Lacking the ability to generate action potentials, astrocytes have classically been thought of as passive cells that only serve to provide support to neurons. However, over the past two decades, astrocytes have been shown to be actively involved in cross talk with neurons [2,3,4]. This interplay is possible since astrocytes express various receptors that are activated by neuronal signals, which subsequently generate calcium waves within astrocytes. Calcium signaling-dependent astrocyte–neuron communication enables astrocytes to be critical mediators of information transfer in our brains [5,6,7]. Indeed, studies have shown that astrocytes are involved in almost every aspect of brain function, and their importance in the central nervous system is reinforced by the fact that all neurological disorders involve some form of dysregulated astrocyte function [8]. Although significant advances have been made regarding how astrocytes contribute to neuronal information processing [9,10,11,12,13,14,15], the gene expression networks that direct astrocyte–neuron communication still remain largely unknown.

In the brain, astrocytes accommodate the local needs of neurons by exhibiting regional specialization. Cortical astrocytes selectively promote synaptic activity of neurons from only the same region [16], and region-specific astrocyte gene expression signatures correlate with neural-circuit-based functional differences [17]. Furthermore, astrocyte transcription factors oversee region-specific astrocyte–neuron communication and subsequent animal behaviors associated with learning and memory [14]. Astrocytes also exhibit region-specific transcription factor expression [18], region-specific astrocyte subtypes [19], and region-specific disease-associated gene signatures of different astrocyte populations [20,21]. These observations of region-specific astrocyte gene expression and function imply that astrocytes respond to the surrounding neuronal environment; critically how these responses are directed by neuron-dependent gene expression changes in astrocytes remains undefined. A recent study has shown that neurons influence gene expression changes in astrocyte Notch signaling that drives neurotransmitter uptake and function [22]; however, whether neuron-dependent changes in astrocyte gene expression affect neurotransmitter and neuromodulator receptors remains unknown. This is a central question in astrocyte biology, because activation of these receptors represents the first step of signal transduction mechanisms that sculpt astrocyte–neuron communication.

Given the vast diversity of neuronal subtypes that densely populate the brain, it is possible that astrocytes exhibit transcriptomic profiles that are calibrated to the type of neuronal signaling that surrounds them. Studies have shown that glutamergic signaling mediated by astrocyte *Grm5* receptor regulates functional maturation of cortical astrocytes [23]. Striatal medium spiny neurons activate astrocytic gamma-aminobutyric acid (GABA)ergic receptors, leading to behavioral and synaptic effects [12]. Cholinergic neuromodulation in the hippocampus inhibits dentate granule cells by activating astrocytes [24] and tunes astrocyte-dependent gating of hippocampal NMDA receptors to wakefulness [25], while noradrenergic neuromodulation has been shown to prime astrocytes toward detecting changes in cortical network activity [26]. These observations show how distinct types of neuronal signaling affect astrocyte function within neural circuits; however, whether different neurotransmitters and neuromodulators control changes in astrocyte gene expression programs remains essentially unknown.

In this study, we developed mouse embryonic stem cell-derived astrocyte cultures to find out how neurons and neuroactive compounds affect astrocyte transcriptomics and chromatin architectures. First, we defined how neurons affect astrocyte gene expression with respect to various neurotransmitter and neuromodulator receptors by RNA-Seq analysis of co-cultures of stem cell-derived mouse astrocytes and human neurons to enable in silico separation of transcriptomic data. Next, we exposed astrocyte cultures to a panel of neuroactive compounds to identify transcriptomic and chromatin accessibility maps in astrocytes, through RNA-Seq and assays for transposase-accessible chromatin (ATAC)-Seq analysis, respectively. We observed that astrocytes display unique transcriptomic responses to each neuroactive chemical and showed that noradrenaline triggers an enhanced effect on astrocytes in comparison to glutamate, GABA, acetylcholine, and serotonin. This is illustrated through enhanced transcriptomic and chromatin accessibility changes in vitro and enhanced calcium signaling in in vivo astrocytes in response to noradrenaline. Together, our analyses define distinct astrocyte gene expression networks that are triggered by neuroactive compounds, identifying noradrenergic signaling as a critical mediator of astrocyte–neuron communication. 

## 2. Results

### 2.1. Generation of Mouse Embryonic Stem Cell-Derived Astrocyte Cultures from 3D Organoid-Like Spheres

**Figure 1:**
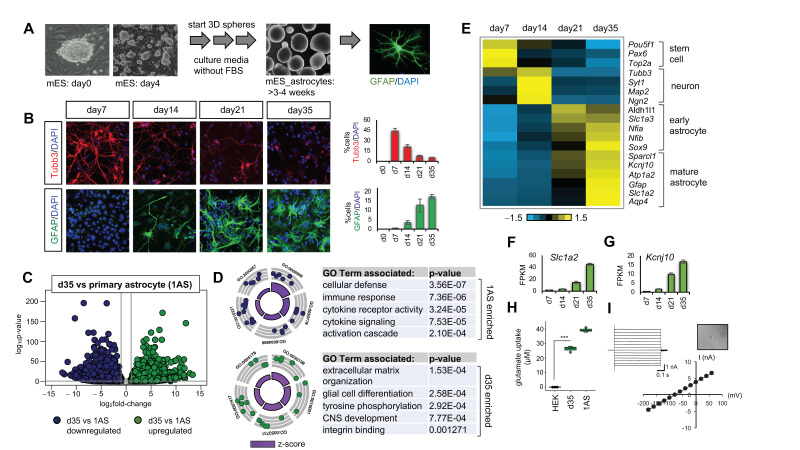
Generation of mouse embryonic stem cell derived astrocytes (mES_astrocytes) from 3D organoid-like spheres. (**A**) Schematic of culture protocol. (**B**) Analysis of Tubb3 and GFAP in mES_astrocyte culture from day 7 to day 35 (*n* = 3 images per time point). (**C**) Volcano plot showing significance versus log_2_fold-change depicting RNA-Seq data comparing serum-containing primary culture (1AS) and day 35 mES_astrocyte transcriptome (*n* = 3, *p* < 0.05, fold-change >2). (**D**) Gene Ontology (GO) circle plot and table showing top GO terms found in differentially expressed genes (DEGs; *p* < 0.01). (**E**) Heatmap showing stem cell and neuronal, early, and later mature astrocyte markers over time in culture. (**F**) Transcript expression of glutamate transporter (*Slc1a2*) and (**G**) potassium channel (*Kcnj10*) over time in mES_astrocytes. (**H**) Kinetics of cellular uptake of L-glutamate normalized to µg protein (*n* = 3 for each group, *** *p* < 0.001, one-way analysis of variance (ANOVA)) of day 35 mES_astrocytes. (**I**) Whole-cell patch clamp electrophysiology of day 35 mES_astrocytes (*n* = 10 cells). Data are shown as the mean ± s.e.m.

We generated astrocytes differentiated from mouse embryonic stem cells (mES_astrocytes) by modifying existing protocols [27]. Briefly, this method involved expansion of mouse embryonic stem cells, generation of 3D organoid-like spheres in a medium that supports neural stem cells for 1 week, and subsequent culture in a differentiation medium. Spheres were maintained by regular dissociation and sphere reformation (Figure 1A). Generation of astrocytes was followed through the use of astrocyte markers NFIA, Sox9, and GFAP; neuronal marker Tubb3; and oligodendrocyte markers MBP and MAG. Loss of neurons was detected after day 7 of culture and reduced to <1% by day 35, whereas GFAP was induced from day 14 and levels increased until day 35 (Figure 1B). Both NFIA and Sox9 were induced between days 7 and 14 (Figure S1A,B) and showed no overlap with Tubb3 (Figure S1C). The presence of oligodendrocytes was also detected after day 14, but these remained at <15% (Figure S1D), and co-labeling with MBP or MAG showed little or no overlap with NFIA or Sox9, which are expressed in >80% of cells (Figure S1A,B).

The fundamental difference between mES_astrocytes and existing in vitro methods of astrocyte generation [27,28,29,30,31,32,33] is the absence of fetal bovine serum (FBS) or ciliary neurotrophic factor (CNTF) in culture media that are known to push astrocytes to a reactive state. Indeed, the addition of FBS/CNTF led to >95% GFAP-positive cells (Figure S1E); however, such GFAP upregulation did not correlate with upregulation of astrocyte functional markers *Aqp4* and *Glt1* (Figure S1F,G). Moreover, comparison of RNA-Seq transcriptomics of day 35 mES_astrocytes and serum-containing primary culture (1AS) astrocytes revealed that 1AS showed significant upregulation in Gene Ontologies (GOs) associated with an inflammatory response similar to a reactive state (Figure 1C).

In contrast, GO terms associated with mES_astrocyte-enriched genes involved structural organization and nervous system development (Figure 1D). Indeed, mES_astrocytes displayed gradual acquisition of mature astrocyte markers over time (Figure 1E), showing that mES_astrocytes represent endogenous astrocyte transcriptomic profiles more accurately. Finally, we showed that mES_astrocytes are functional by testing two aspects associated with mature astrocyte function: glutamate transport and passive conductance. mES_astrocytes expressed increasing levels of glutamate transporter Glt1 (*Slc1a2*) and potassium channel (*Kcnj10*) over time from day 7 to day 35 (Figure 1F,G). We also showed that mES_astrocytes have glutamate uptake ability (Figure 1H) and passive conductance (Figure 1I).

### 2.2. Co-Culture with Neurons Induces the Expression of Neurotransmitter and Neuromodulator Receptor Subtypes in mES_astrocytes

**Figure 2:**
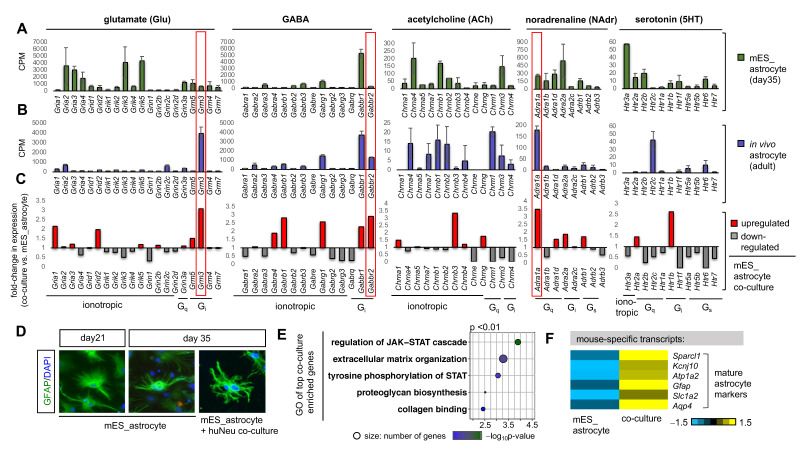
Neurons trigger the expression of distinct receptor subtypes in astrocytes and promote astrocyte development. Transcript expression levels of different receptor subtypes from RNA-Seq data of (**A**) day 35 mES_astrocytes and (**B**) 3–5-month-old adult astrocytes purified by FACS from Aldh1l1-GFP mice (*n* = 3). Data are shown as the mean ± SD. (**C**) Average fold-change in the transcript expression of receptor subtypes in mES_astrocyte co-cultures versus mES_astrocyte control cultures. In vivo enriched receptor subtypes that are triggered by neuronal contact are highlighted in red boxes. (**D**) mES_astrocytes in co-cultures with neurons become morphologically complex in comparison to control cultures, as observed by GFAP immunolabeling. (**E**) GO terms of top genes that are enriched in co-cultures (*p* < 0.01). (**F**) Heatmap showing that mature astrocyte markers are enriched in mES_astrocyte co-cultures in comparison to control mES_astrocyte monocultures.

Having established that mES_astrocytes more accurately reflect the molecular properties of healthy in vivo astrocytes, we next evaluated the expression of key receptors of neurotransmitters and neuromodulators in this system. mES_astrocytes abundantly expressed receptors for various types of neuronal signaling involving glutamate (Glu), GABA, acetylcholine (ACh), noradrenaline (NAdr), and serotonin (5HT) (Figure 2A). However, the relative levels of different receptor subtypes mostly differed from those of in vivo astrocytes [14], especially with respect to receptors *Grm3* (Glu), *Gabbr2* (GABA), *Adra1a* (NAdr), and *Htr2c* (5HT) (Figure 2B). Moreover, receptor expression levels of 1AS cultures largely differed from expression profiles of both in vitro mES_astrocytes and in vivo astrocytes (Figure S2A).

Since endogenous astrocytes reside in an interconnected network with neurons, we next asked how the addition of neurons to mES_astrocytes would influence relative receptor expression. We co-cultured mES_astrocytes with human stem-cell-derived neurons (huNeu), allowing species-based in silico separation of mouse astrocyte and human neuron transcripts [22] to bypass physical purification of astrocytes. Human neurons were derived from induced pluripotent stem cells [34], added to day 21 mES_astrocytes, and maintained for 2 weeks until day 35. RNA-Seq of astrocyte co-cultures in comparison to controls revealed that neurons indeed trigger the expression of endogenously enriched receptors for *Grm3*, *Gabbr2*, and *Adra1a* in mES_astrocytes (Figure 2C). Moreover, we observed noticeable morphological changes in astrocytes in the presence of neurons (Figure 2D), since direct contact with neuronal processes drives astrocyte morphogenesis [35]. Overall, neuronal influence on mES_astrocytes directed astrocyte maturation, as demonstrated by enrichment of GO terms associated with the JAK-STAT pathway (Figure 2E), which is essential for astrocyte development and upregulation of mature markers (Figure 2F and Figure S2B). Together, these results show that mES_astrocytes can respond to neuronal contact, which drives astrocyte development and triggers the expression of distinct receptor subtypes in mES_astrocytes.

### 2.3. Identification of Neuron-Dependent Astrocyte Maturation Gene Signatures

**Figure 3:**
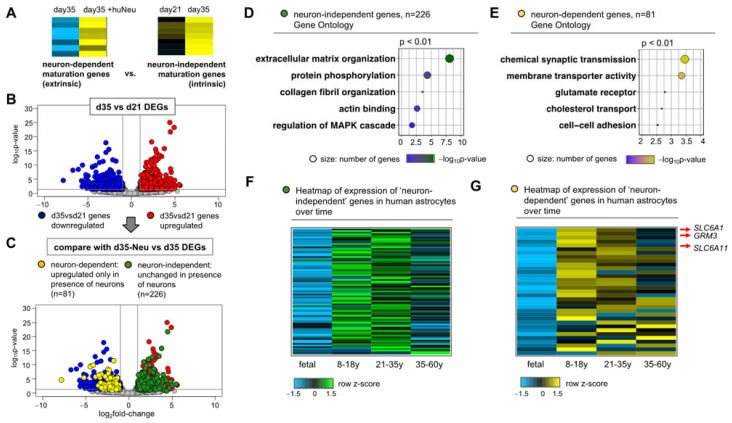
A mixed-species co-culture system enables the identification of neuron-independent and neuron-dependent programs of astrocyte development. (**A**) Schematic of the bioinformatic approach to identify gene sets. (**B**) Volcano plot showing significance versus log_2_fold-change depicting RNA-Seq data comparing day 35 and day 21 mES_astrocyte transcriptomes (*n* = 3, *p* < 0.05, fold-change > 2). (**C**) Comparison of DEGs shown in (**B**) with genes differentially expressed in co-cultures to identify neuron-independent and neuron-dependent genes, shown by green and yellow circles, respectively. (**D**,**E**) GO terms of top genes from both sets (*p* < 0.01). Heatmap showing expression levels of (**F**) neuron-independent and (**G**) neuron-dependent gene sets in human astrocytes at different ages, from fetal to 60 years. Detailed gene lists used for heatmap generation are given in Table S1.

The above observations suggest that the addition of neurons to astrocyte cultures stimulates their maturation, creating a venue by which we can decipher neuron-dependent and neuron-independent developmental programs in an in vitro model of differentiating astrocytes. The time window of 2 weeks between day 21 and day 35 in mES_astrocytes is representative of rapidly developing astrocytes (Figure 1E), and this maturation phase is accelerated by co-culture with neurons (Figure 2E,F). With the help of these two culture systems of mES_astrocytes days 21–35 and mES_astrocyte/huNeu co-culture, we interrogated neuron-dependent and neuron-independent gene expression programs during astrocyte maturation (Figure 3A).

We identified differentially expressed genes (DEGs) by RNA-Seq analysis of day 35 mES_astrocytes compared to day 21 astrocytes, identifying 412 upregulated and 457 downregulated DEGs (Figure 3B). GO terms associated with the 412 DEGs upregulated at day 35 include structural and signal-transduction-related terms (Figure S3A). We next asked how many of these day 35 upregulated DEGs are unchanged in day 35 co-cultures with neurons, revealing a subset of 226 genes (Figure 3C). This subset represents genes that are upregulated during astrocyte maturation and are intrinsic to astrocyte development independent of neuronal influence. GO terms associated with the neuron-independent subset are related to structural and signal transduction genes (Figure 3D). Conversely, we also asked how many of day 35 downregulated DEGs are upregulated in day 35 co-cultures with neurons, revealing a subset of 81 genes (Figure 3C), which represents astrocyte development genes extrinsic to astrocytes and neuronal dependent. Not surprisingly, GO terms associated with the neuron-dependent subset include synaptic transmission and neurotransmitter receptor genes (Figure 3E). In addition, we identified 229 genes that are not significantly differentially expressed between day 21 and 35 mES_astrocytes but are upregulated in the presence of neurons in co-culture. These also fall under neuron-dependent astrocyte development genes since they require neuronal presence for increased expression (Figure S3B–D).

Next, we asked whether the neuronal-associated maturation signatures identified above are present in human astrocytes. Using transcriptomic data from human astrocytes at different ages, from fetal to 60 years of age [36], we identified a subset of ~50 neuron-independent genes that are enriched at least twofold in both age groups of 8–18 years and 21–35 years over fetal astrocytes (Figure 3F). Within the two age groups, 8–18 years and 21–35 years, only 8 genes were enriched in the 21–35-year age group, indicating that once astrocytic neuron-independent maturation genes are established, their expression levels are maintained at consistent levels till 18–35 years of age. Interestingly, a majority of these genes show age-dependent decline by 60 years of age. Similar trends were observed for the neuron-dependent gene sets (Figure 3G and Figure S3D). Detailed lists of neuron-independent and neuron-dependent gene expression programs relevant during human astrocyte maturation are given in Table S1. 

Since regional astrocyte heterogeneity adapts to local neuronal circuits [14,17], we also asked whether neuron-dependent genes are enriched in brain region-specific genes [18], revealing subsets of neuron-dependent genes that are differentially upregulated in the cortex, olfactory bulb, and brainstem (Table S1). We also asked how astrocyte synapse-associated genes [37] are affected by the presence or absence of neurons. Of the astrocyte-secreted synaptogenic factors, Thbs2 from the thrombospondin family is a neuron-independent astrocyte maturation gene whereas Gpc5 from the glypican family and Sparcl1 are neuron-dependent astrocyte maturation genes. Synapse maturation gene Chrdl1 [38] and synapse elimination genes Megf10 and Mertk [39] are not affected by neurons in co-cultures or upregulated in astrocyte monocultures from day 21 to day 35 (Figure S2C). Given the short time period of 2 weeks in neuron co-culture, it is likely that the astrocyte transcriptomic changes induced upon neuronal contact are not due to synapse–astrocyte interactions. Overlap of neuron-dependent genes with previously published neuron-induced astrocyte transcriptomic changes [22] are given in Table S1. 

Analysis of the neuron-independent and neuron-dependent gene sets for various receptors (shown in Figure 2A,C) showed that neuron-independent genes are not enriched for receptors but neuron-dependent astrocyte development genes contain receptors for Glu (*Grm3*), NAdr (*Adra1a*), and also GABA transporters *Slc6a1*/*Slc6a11* (Figure 3G and Figure S3C). This shows that co-culture systems can be exploited to identity distinct receptor subtypes that are dependent on neuronal contact for astrocyte development.

### 2.4. Neuroactive Compounds Induce Broad Changes in Astrocyte Expression Profiles and Chromatin Accessibility Landscapes

**Figure 4:**
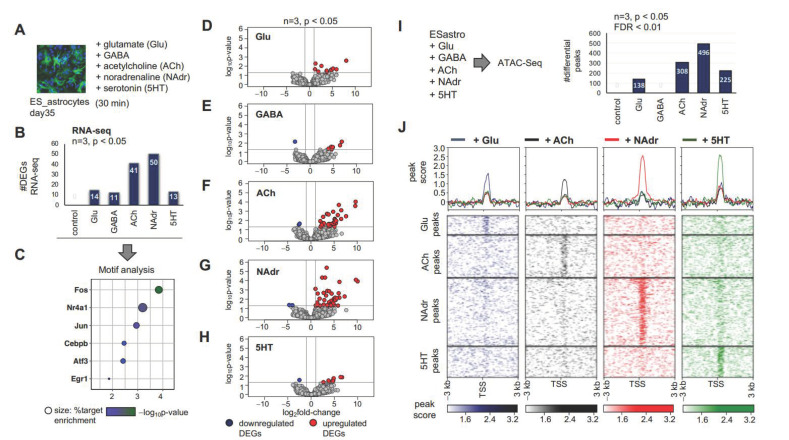
Neuroactive chemical-induced gene regulation in mES_astrocytes. (**A**) Schematic of stimulation of mES_astrocytes by a panel of neuroactive chemicals. (**B**) Bar graph showing the total number of DEGs from RNA-Seq for each chemical (*n* = 3, *p* < 0.05, fold-change > 2). DEG lists associated with each chemical are given in Table S2. (**C**) Significant transcription factor motifs (*p* < 0.01) enriched in all DEGs shown in (B). (**D**–**H**) Volcano plots showing significance versus log_2_fold-change depicting RNA-Seq data comparing transcriptomes in response to (**D**) Glu, (**E**) GABA, (**F**) ACh, (**G**) NAdr, and (**H**) 5HT (*n* = 3, *p* < 0.05, fold-change > 2). (**I**) Schematic of assays for transposase-accessible chromatin (ATAC)-Seq experiment and bar graph showing the total number of genes with differential peaks for each chemical (*n* = 3, *p* < 0.05, FDR for peak enrichment < 0.01). (**J**) Heatmap of ATAC-Seq analysis depicting chromatin peaks that become accessible in response to chemicals. Peaks within 3 kb of transcription start sites are shown (*n* = 3, *p* < 0.01).

Since neuronal contact regulated the expression of key neurotransmitter and neuromodulator receptors on astrocytes, we next determined how specific neuron-derived signals influence gene expression profiles in astrocytes. We used a panel of five neuroactive compounds (glutamate (Glu), gamma-aminobutyric acid (GABA), acetylcholine (ACh), noradrenaline (NAdr), and serotonin (5HT)) to ask how different types of neuronal-derived signals affect transcriptomic profiles in astrocytes. Day 35 mES_astrocytes were exposed to this panel for 30 min (Figure 4A), and RNA-Seq analysis showed transcriptomic changes in response to these cues (Figure 4B). Motif analysis of the DEGs induced by this panel identified well-established immediate early genes, like Fos, Jun, Egr1, Atf3, Nr4a1, and Cebpb (Figure 4C). These are known to be induced in response to neuronal activity [40], suggesting that the addition of neuron-derived signals to mES_astrocytes similarly modeled immediate early responses previously identified in neurons. Volcano plots of mES_astrocyte DEGs in response to the different chemical cues (Figure 4D–H, Table S2) showed that NAdr (Figure 4G) induces a more robust change in mES_astrocyte transcriptomics than Glu (Figure 4D), GABA (Figure 4E), ACh (Figure 4F), or 5HT (Figure 4H).

Given the changes in gene expression induced by these neuroactive compounds, we next evaluated how they influenced the chromatin accessibility landscape of astrocytes by performing assays for transposase-accessible chromatin (ATAC) and sequencing to assess genome-wide chromatin accessibility maps [41]. Since relaxation of chromatin architecture precedes transcription, this provided a broader scope to determine how neuronal chemicals transform the transcriptional landscape of astrocytes. As with RNA-Seq, we observed robust changes in chromatin architectures in response to different neuroactive compounds with the exception of GABA. Furthermore, similar to RNA-Seq data, we observed that NAdr induced enhanced chromatin accessibility in mES_astrocytes in comparison to Glu, GABA, ACh, or 5HT (Figure 4I). This result is also reflected in the heatmap of ATAC-Seq data, wherein NAdr shows stronger peak scores over a larger number of chromatin-accessible sites (Figure 4J). Notably, although 5HT showed a lower number of peaks, it displayed peak scores similar to those of NAdr, implying stronger effects albeit at a lower number of chromatin-accessible sites.

**Figure 5:**
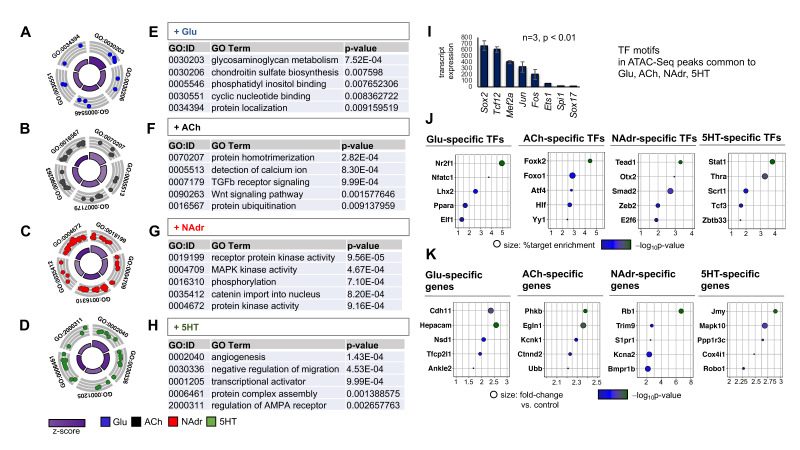
Unique chromatin accessibility signatures are exhibited by mES_astrocytes in response to different neuroactive chemicals. (**A–H**) GO circle plot and tables showing top GO terms found in DEGs (*p* < 0.01) associated with each chemical: (**A**,**E**) Glu, (**B**,**F**) ACh, (**C**,**G**) NAdr, and (**D**,**H**) 5HT. Note that NAdr induces the most differentially enriched chromatin-accessible sites across the panel of chemicals. (**I**) Significant transcription factor motifs (*p* < 0.01) enriched in all differential ATAC-Seq peaks from Glu, ACh, NAdr, and 5HT. Transcription factors are shown based on transcript expression levels in in vivo astrocytes. (**J**,**K**) Top five (**J**) transcription factor motifs that are enriched at chromatin-accessible peaks and (**K**) genes with open chromatin signatures that are induced unique to each neurochemical in comparison to controls (*n* = 3, *p* < 0.05) and filtered based on transcript expression levels of in vivo astrocytes. Detailed lists of these genes are given in Table S4.

We next examined how NAdr induces higher numbers of transcriptomic and chromatin architectural changes in mES_astrocytes. First, we analyzed the expression of different receptors for Glu, GABA, ACh, NAdr, and 5HT and observed no significant differences after exposure of mES_astrocytes to the panel of neuroactive compounds (Figure S4A–E). Next, we evaluated the functional ontologies of genes associated with the open chromatin peaks induced by each compound, excluding GABA from this analysis since we failed to see significant enrichment of chromatin accessibility. Similar to observations made in Figure 4I,J, NAdr induced chromatin accessibility at a larger number of genes compared to other chemical cues, as shown in GO plots (Figure 5A–D). Analysis of top GO terms showed that each neuronal chemical cue transformed mES_astrocyte chromatin accessibility in distinct ways (Figure 5E–H), and NAdr induced chromatin accessibility at genes associated with signal transduction pathways involving protein kinase activity cascades and protein phosphorylation (Figure 5G), such as *Prkd1*, *Prkd2*, *Prkacb*, *Camk4*, *Map3k13*, and *Pik3cg* (Table S3).

We used the chromatin accessibility signatures associated with each neuroactive chemical to define distinct sets of gene regulatory networks unique to each. First, we performed transcription factor motif analysis of chromatin-accessible peaks shared by the panel of chemicals tested. This revealed calcium-induced transcription factors such as Fos and Jun, as expected (Figure 5I). Next, we analyzed chromatin-accessible peaks unique to each neuroactive chemical to identify distinct sets of transcription factor motifs induced specifically by Glu, ACh, NAdr, or 5HT. Results from this analysis revealed nuclear receptor Nr2f1, forkhead box protein Foxk2, transcriptional enhancer Tead1, and STAT family protein Stat1 as among the most enriched binding motifs exclusive to Glu, ACh, NAdr, or 5HT, respectively (Figure 5J). Similarly, we identified which genes become chromatin accessible following astrocyte stimulation by Glu, ACh, NAdr, and 5HT and identified gene sets distinct for each neuroactive chemical (Table S4). The most statistically enriched genes identified were potassium channel *Kcnj1*, trophic factor *Ndnf,* actin-interacting protein gene *Plekho1*, and extracellular matrix protein gene *Reln*, exclusive to Glu, ACh, NAdr, or 5HT, respectively (Figure S5). Interestingly, the NAdr-specific gene *Plekho1* is known to be involved in protein phosphorylation cascades by facilitating phosphorylation of actin and may explain the strong induction of signal transduction pathways observed for NAdr. We further filtered each neuroactive chemical exclusive gene set based on the transcript expression level of in vivo astrocytes [14]. The top 20 genes were taken from each gene set and rearranged based on astrocyte-specific transcript levels, and the top genes identified were cadherin *Cdh11*, phosphorylase kinase *Phkb*, transcriptional corepressor *Rb1*, and junction protein *Jmy*, exclusive to Glu, ACh, NAdr, and 5HT, respectively (Figure 5K). These observations demonstrated that each neuroactive chemical affect astrocyte chromatin accessibility in unique ways and the effects induced by NAdr are more enhanced than others.

### 2.5. NAdr Induces Heightened Astrocytic Calcium Responses In Vivo

**Figure 6:**
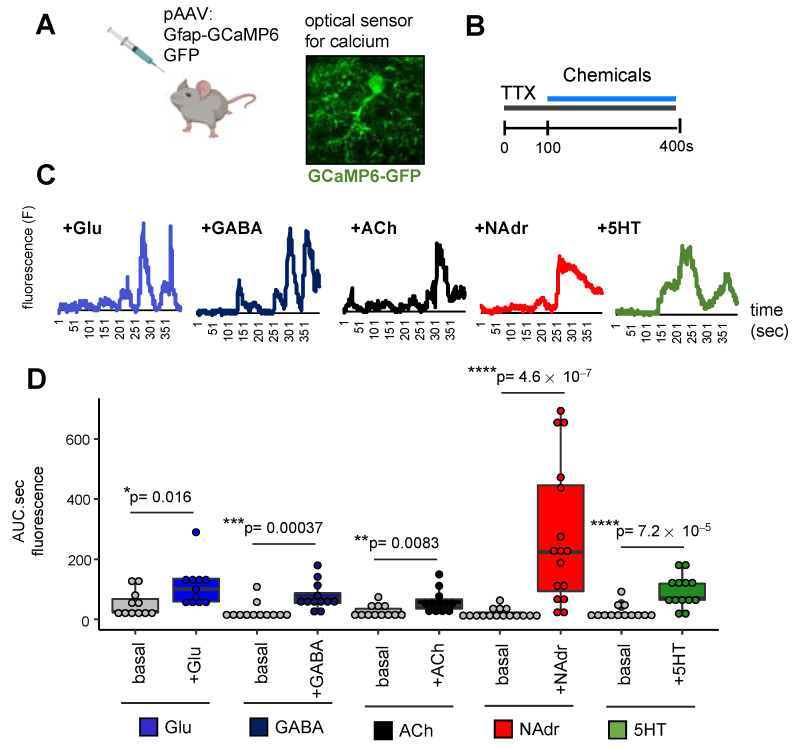
NAdr induces enhanced calcium signaling in vivo compared to Glu, GABA, ACh, and 5HT. (**A**,**B**) Schematic of calcium imaging experiment showing (**A**) expression of calcium sensor and (**B**) experimental paradigm of neurochemical exposure. (**C**) Representative calcium traces from single astrocytes in response to Glu, GABA, ACh, NAdr, and 5HT. (**D**) Quantification of area under the curve from stimulated astrocytes in comparison to basal (*n* = 4–5 mice, 11–16 cells per sample, * *p* < 0.05, ** *p* < 0.01, *** *p* < 0.001, **** *p* < 0.0001, Wilcoxon test).

The above transcriptomic and chromatin accessibility profiling studies suggest that mES_astrocytes are more responsive to NAdr than other neuroactive chemicals we examined. Therefore, we next evaluated whether astrocytes in vivo demonstrate differential responses to this cohort of neuroactive compounds by measuring calcium activity in response to these compounds. Toward this purpose, we used adenoviral vector (pAAV) to deliver a fluorescent optical sensor for calcium, GCaMP6-GFP [42], under the control of the *Gfap* promoter for astrocyte-specific expression (Figure 6A) of the calcium sensor.

Brain slices were prepared from 3-month-old adult mice expressing GCaMP6-GFP and treated with TTX to block neuronal electrical activity before application of Glu, GABA, ACh, NAdr, or 5HT for 100–400 s (Figure 6B). Two-photon imaging of calcium-dependent fluorescence showed that astrocytes ex vivo responds to different neuronal chemical cues, as shown by representative traces from a single astrocyte (Figure 6C). It is to be noted that these are representative traces of single astrocytes, and variation in oscillation patterns were observed across traces from different astrocytes. Overall, we did not observe oscillation patterns unique to each neuroactive chemical. Quantification of total fluorescence from all cells in response to Glu, GABA, ACh, NAdr, or 5HT in comparison to basal levels in the absence of application of these chemicals revealed calcium levels to be dramatically elevated in response to NAdr compared to those in response to Glu, GABA, ACh, or 5HT. Though each neuronal chemical cue induced a statistically significant calcium response in astrocytes (*p* < 0.05, Wilcoxon test), the effect of NAdr was greater than that of Glu, GABA, ACh, or 5HT (Figure 6D and Figure S6A). Apart from NAdr, both GABA and 5HT induced higher levels of calcium than Glu or ACh. Further comparison to the Glu-induced calcium response showed that only 5HT triggered a statistically significant calcium response over GABA or ACh (*p* < 0.05, Wilcoxon test, Figure S6B). Taken together, these studies provide an entry point for exploring how different types of neuronal signaling affect astrocyte function and how dysregulated neuronal signaling may contribute to disease through these gene regulatory programs.

## 3. Discussion

### 3.1. An In Vitro Transcriptomic Approach to Studying Gene Regulatory Programs of Astrocyte Neuron Communication

Astrocytes are integral components of neuronal circuits and are actively involved in information processing by the brain [8]. Although tremendous advances defining the role of astrocytes in cross talk with neurons have been made, detailed mechanisms with respect to gene expression programs associated with astrocyte–neuron communication have remained elusive. Here, we used an in vitro system to define transcriptomic and chromatin accessibility programs in astrocytes that are stimulated by neurons and neuroactive chemicals. To circumvent the drawbacks associated with using serum, we used a serum-free in vitro culture system of astrocytes derived from mouse embryonic stem cells (Figure 1), and a co-culture with neurons provided a route to drive astrocyte maturation with respect to gene expression in vitro (Figure 2). A recent study reported the use of in vitro culture system to show that neuron-derived factors trigger ryanodine-receptor-mediated calcium-induced calcium release, a phenomenon well studied in neurons but previously undocumented in astrocytes [43]. Here, we used these in vitro culture systems to ask two questions that are extremely difficult to address in vivo using current technologies: (1) What are neuron-independent and neuron-dependent astrocyte maturation gene expression programs? (2) How do different types of neuronal signaling affect astrocyte transcriptomics and chromatin architecture? Our studies (1) identified neuronal-contact-dependent changes in astrocyte transcriptomics with respect to receptor subtype expression and (2) established that noradrenergic in comparison to glutamatergic, GABAergic, cholinergic, and serotonergic signaling induces enhanced effects in astrocytes both in vitro and in vivo in the adult mouse brain.

### 3.2. Neuronal Contact Is Necessary for the Expression of Distinct Receptor Subtypes in Astrocytes and Promotes Astrocyte Development

A recent study using astrocytes purified by immunopanning and cultured in vitro showed that secreted signals from microglia, oligodendrocyte precursors, or endothelial cells did not change astrocyte maturation [33]. This finding implies that contact with neurons, as shown in this study, but not with other cell types of the brain, may provide an efficient method to generate more mature astrocytes in vitro. Indeed, human astrocyte–neuron co-cultures generate structurally complex astrocytes with increased density of synapses [34]. An earlier study used mouse and rat astrocyte–neuron co-cultures to define how neurons induce Notch signaling in astrocytes to drive maturation and neurotransmitter uptake function in astrocytes [22]. Here, we extended this concept to identify neuron-independent and neuron-dependent gene expression programs in astrocytes. This is difficult to address in vivo since the birth of astrocytes occurs after the birth of neurons, hindering identification of genes intrinsic to astrocyte maturation. On the other hand, neuronal synaptogenesis and maturation begin only after astrocytes are born, raising questions regarding how extrinsic neuron-triggered transcriptomic changes in astrocytes may further trigger synapse maturation. Here, we dissected distinct sets of gene expression programs that are intrinsic or extrinsic to astrocyte development (Figure 3A–E). We found that the glutamate receptor *Grm3*, GABA transporters *Slc6a1*/*Slc6a11*, and the noradrenergic receptor Adra1a are extrinsic (neuron-dependent) to astrocyte development, revealing how the neuronal-induced astrocytic expression of signaling components might influence synapse maturation in the developing brain. It is to be noted that although the Ngn2-induced neurons used in this study are glutamergic, changes induced by glutamatergic neurons with respect to GABAergic signaling and other neuromodulatory signaling components in astrocyte transcriptomes are unexpected and reveal the existence of potential cross-signaling mechanisms in astrocyte–neuron interactions. Furthermore, we also identified subsets of these extrinsic and intrinsic gene expression programs that are elevated during human brain development, and interestingly, a majority of these genes show a decline in the ageing human brain (Figure 3F,G, Table S2).

### 3.3. Neuroactive Compounds Affect the Expression of Potassium Channels

A key result of interest was the observation that astrocyte development and stimulation with neuroactive chemicals induced the expression of inwardly rectifying potassium channels (Kir): astrocyte development from day21–35 promoted the expression of *Kcnj14* and neurotransmitter glutamate induced *Kcnj1* expression. The expression levels of Kir channels would essentially affect potassium homeostasis, which is especially important with regard to potassium clearance following neuronal activity. Potassium clearing by astrocytes has been shown to improve the signal-to-noise ratio of synaptic transmission, and astrocyte-mediated control of extracellular potassium levels is a powerful mechanism of modulation of network activity [44]. The importance of astrocytic Kir channels [45] is further demonstrated by findings that showed that astrocyte Kir4.1 channel deficit contributed to neuronal dysfunction in a mouse model of Huntington’s disease [46].

### 3.4. Noradrenergic Signaling Is an Important Mediator of Astrocyte–Neuron Communication

Neuronal activity encompasses electrical firing in neurons, wherein different subtypes of neurons release different types of chemicals to transmit signals. Neurotransmitters Glu and GABA are released by glutamergic and GABAergic neurons, respectively, and are extensively distributed throughout the brain. Neuromodulators like ACh, NAdr, and 5HT enhance the effects of neurotransmitters and modulate larger sets of neurons. Cholinergic neurons originate from the basal forebrain and the brainstem, while noradrenergic and serotonergic neurons are from the locus coerulus and dorsal raphe nuclei of the brainstem, respectively, and project processes that densely permeate the entire brain. Astrocytes, forming tripartite synapses, are active members of this neuronal pool of chemicals involved in information transfer. However, whether different kinds of neuronal signaling differentially affect astrocyte genetic programs that lead to synaptic control is unknown. This is largely due to the fact that manipulation of neuronal activity involving chemogenetic (DREADD) or optogenetic (ChR2) approaches is driven by pan-neuronal promoters [47] and manipulation of neuronal activity of cholinergic, noradrenergic, or serotonergic neurons requires the expression of ChR2 or DREADDs at specific neuronal subtypes [24,25,48]. Furthermore, studies involving direct activation of astrocytes [9,10,11,12,13,49,50] also do not reflect how different types of neuronal signaling affect astrocytes differentially.

On the other hand, in vitro methodologies to study the effects of neuronal activity use either pharmacological agents, such as bicuculline [22], or ChR2 using pan-neuronal promoters [51]. Dissecting specific effects of each type of neuronal signaling would require systematic co-culture with each neuronal subtype that would require differential culture conditions. Here, we used a simplified system to apply these different chemical cues to mES_astrocytes for a short time period to model different types of neuronal signaling. Indeed, the detection of binding motifs for known neuronal-activity-induced transcription factors such as Fos and Jun revealed that this system recapitulates an environment of neuronal activity surrounding astrocytes (Figure 4C).

We expected that different neuroactive chemicals would largely evoke similar responses in astrocytes. In contrast, our results show that different kinds of neuronal signaling induce predominantly different sets of transcriptomic changes in astrocytes, which is also reflected at the level of global chromatin accessibility maps (Figure 4D–H,J). Surprisingly, the inhibitory neurotransmitter GABA failed to induce statistically significant (significant *n* = 3, *p* < 0.05, FDR < 0.01) chromatin accessibility changes in mES_astrocytes in comparison to Glu, ACh, NAdr, or 5HT. However, in vivo studies involving the activation of Gi-DREADDs in striatal astrocytes have shown robust changes in astrocyte transcriptomics, with behavioral outputs associated with hyperactivity and disrupted attention [12]. This shows that recapitulating effects of inhibitory neurotransmission on astrocytes may not be feasible using in vitro methods.

Furthermore, NAdr induced the most dramatic effects in both in vitro and in vivo astrocytes (Figure 4 and Figure 6). This effect of noradrenergic signaling is due to enhanced signal transduction pathways, and a similar mechanism is likely reiterated in vivo, since NAdr induced elevated levels of calcium, which is upstream of signal transduction pathways. In addition, in vivo studies of mouse behavior and calcium signaling have shown that NAdr, and not Glu or ACh, is the primary mediator of astrocyte calcium signaling in the adult cortex [52]. During locomotion, noradrenaline triggers calcium signaling in astrocytes to prime astrocyte response to local neuronal activity [26]. Furthermore, our result that NAdr triggers elevated calcium levels in comparison to glutamergic signaling is consistent with observations that Glu does not trigger calcium signaling in the adult cortex in mouse [53], due to predominance of the *Grm3* glutamate receptor, which is coupled to cyclic AMP signaling instead of calcium. This shows that simple in vitro culture systems can provide effective means to study details of astrocyte–neuronal communication at the level of different neuronal subtypes. Indeed, we used the chromatin accessibility datasets generated for this study to define transcription factor binding motifs and gene expression programs exclusive to different types of neuroactive chemicals (Figure 5, Table S4). These datasets will be of fundamental importance in future studies elucidating astrocyte function in response to distinct types of neuronal signaling in the healthy brain and how these are dysregulated in brain disease.

## 4. Materials and Methods

### 4.1. Animals

All research and animal care protocols were in accordance with the US Department of Health and Human Service, National Institute of Health guidelines, and Baylor College of Medicine IACUC guidelines (protocol number AN-5162 approved on 10/30/2020). Mice were housed with food, water, and nesting material in 12 h/12 h light/dark cycles in the Association for Assessment and Accreditation of Laboratory Animal Care-approved animal facility at the Baylor College of Medicine. Both male and female mice of ICR-CD1 background were used for experiments.

### 4.2. Generation of mES_astrocytes and Primary Culture Astrocytes

mES_astrocytes were derived from the mouse embryonic stem cell line ES-D3 (ATCC, Cat. No. CRL-1934, Manassas, VA, USA). Cells were maintained in the stem cell stage on mouse embryonic fibroblast feeder (Fisher, Cat. No. A34181, Waltham, MA, USA) layers on gelatin-coated plates in media comprising DMEM-F12/Glutamax, 15% knockout serum (KOSR, a serum-free formulation; Fisher, Cat. No. 10828-010, USA), 1% non-essential amino acids, 100 µM beta-mercaptoethanol, and 1× leukemia inhibitory factor (Millipore, Cat. No. ESG1106, Burlington, MA, USA). After 3–5 days, stem cells were lifted from the feeder layer using a short 2 min incubation with accutase at 37 °C and introduced to embryoid body (EB) media that promote the growth of neural stem cells in 3D organoid-like spheres. The EB media contained DMEM-F12/Glutamax, 10% KOSR, 1× N2, 1× B27, 100 µM beta-mercaptoethanol, and 1× chemically defined lipid concentrate, and cells were maintained as spheres for 1 week, with media changes in between. After 1 week, spheres were moved to differentiation media that promoted the differentiation of neural stem cells toward astrocytes. The differentiation media contained DMEM-F12/Glutamax, 1× N2, and 1× B27 and were supplemented with growth factors: 10 ng/mL of EGF (Peprotech, Cat. No. 315-09, Cranbury, NJ, USA) and 10 ng/mL of FGF2 (Peprotech, Cat. No. 100-18B, USA). Comparative studies to study the effect of FBS or CNTF used differentiation media containing 1% FBS (Fisher, Cat. No. 16000044, USA) or 10 ng/mL of CNTF (Peprotech, Cat. No. 450-13, USA). We performed all our analyses between 3 and 5 weeks, but the spheres could be maintained in a healthy state with no loss in survival for up to 7–10 weeks. The spheres were maintained with regular dissociation and reformation of spheres in differentiation media. All cultures were maintained in 5% CO_2_ at 37 °C. The above protocol is a modified version of published work generating human astrocytes from induced pluripotent stem cells [27].

For primary astrocytes, dissociated tissue from P0–P2 postnatal brains were cultured on poly-D-lysine-coated flasks in DMEM-F12, FBS, and penicillin–streptomycin. The media were changed the next day, and flasks were vigorously shaken overnight at room temperature to enrich for astrocytes. Cultures were grown for additional 1–2 days before analysis or further passaging. Astrocytes were harvested using trypsin for 5 min at 37 °C. All cultures were maintained in 5% CO_2_ at 37 °C.

### 4.3. Glutamate Uptake Assay and Passive Conductance of mES_astrocytes

Glutamate transport was measured by the ability of astrocytes to clear glutamate from the culture media using the Glutamine/Glutamate Determination Kit (Sigma, St. Louis, MO, USA). Day 35 mES_astrocytes spheres were dissociated using accutase and plated on Matrigel-coated 48-well plates at 20,000 cells per well. The next day, astrocytes were equilibrated in modified HBSS buffer (140 mM NaCl, 4 mM KCl, 2 mM MgCl_2_, 1 mM CaCl_2_, 23 mM glucose, 15 mM HEPES; pH 7.4) for 10–30 min, following which 50 µM L^−^glutamate or 0 µM L^−^glutamate (blank) was added for 1 h. Glutamate levels remaining in the media were measured at 340 nm absorbance following manufacturer instructions of enzymatic assays. The decrease in glutamate in the media (or glutamate uptake) was reported after normalizing to total protein in each well. Protein concentration was determined by Bradford assay (BioRad, Hercules, CA, USA). HEK293 cells and primary astrocytes were used as negative and positive controls, respectively.

For passive conductance measurement, whole-cell recordings were made from mES_astrocytes dissociated with accutase and cultured on Matrigel-coated plates. The holding potential was −70 mV. Pipette resistance was typically 5~8 MΩ, and the pipette was filled with an internal solution of 140 mM K-gluconate, 10 mM HEPES, 7 mM NaCl, and 2 mM MgATP adjusted to pH 7.4 with CsOH. Electrical signals were digitized and sampled at 50 μS intervals with a Digidata 1440A and Multiclamp 700B amplifier (Molecular Devices) using pCLAMP 10.7 software. Data were filtered at 2 kHz.

### 4.4. Generation of Human Neurons and Co-Culture with mES_astrocytes

A transgenic human pluripotent stem cell line was used to derive human neurons (iNeurons), as described previously [34,54], and based on the doxycycline-inducible expression of neurogenin 2 to drive neuron generation. Dissociated day 21 mES_astrocytes were added to doxycycline-induced neurons and grown as mixed neuron–astrocyte spheres for a period of 2 weeks till day 35 in neural media containing DMEM/F12, 1× N2, and 1× B27. Single cultures of neurons or astrocytes maintained in parallel for 2 weeks in the same culture media were used as controls. For both control and co-cultures, 3–5 million cells of each cell type were used.

### 4.5. Immunocytochemistry

Spheres were dissociated using accutase and plated on Matrigel-coated slides. Cells were allowed to grow in differentiation media without EGF+FGF2 for 3–4 days, followed by fixing in 4% PFA for 30 min at 4 °C. After PBS washes, the cells were blocked with 0.25% Triton-X100, 5% donkey serum, and 5% goat serum for 30 min at room temperature, following which the cells were incubated with primary antibodies overnight at 4 °C. The next day, the cells were washed with PBS thrice, followed by incubation with secondary antibodies in PBS containing 1% goat serum and 1% donkey serum for 1 h at room temperature in the dark. After 10 min of incubation with DAPI, the cells were washed with PBS twice and placed under coverslips using Fluoromount-G (Southern Biotech, Birmingham, AL, USA). The following primary antibodies were used: anti-mouse tubulin 3 (1100; Covance, Cat. No. MMS435P, Princeton, NJ, USA), anti-mouse GFAP (1:1000; Millipore, Cat. No. MAB360, Burlington, MA, USA), anti-rabbit NFIA (1:500; Sigma, Cat. No. HPA006111, St. Louis, MO, USA), anti-rabbit Sox9 (1:500; Abcam, Cat. No. AB5535, Cambrige, MA, USA), anti-rabbit MBP (1:500; Sigma, Cat. No. HPA04922, USA), and anti-mouse MAG (1:500; Millipore, Cat. No. MAB1567, USA). The following secondary antibodies were used at 1:500 dilution: Alexa Fluor 568 goat anti-rabbit (Thermo Fisher Scientific, A11036, Waltham, MA, USA) and Alexa Fluor 488 goat anti-mouse (Thermo Fisher Scientific, A11001, USA). Images were acquired via a Zeiss M1 epifluorescent microscope or a Carl Zeiss LSM800 confocal microscope with Zen2.3 software. Image quantification was performed using ImageJ software.

### 4.6. RNA Isolation and RT-qPCR

Spheres frozen in TRIzol (Thermo Fisher, Waltham, MA, USA) were thawed and vortexed for 2 min, followed by chloroform extraction of RNA, which was further purified by the RNeasy Mini Kit (Qiagen, Germantown, MD, USA). Subsequent conversion to cDNA was performed using the iScript Reverse Transcription Supermix (BioRad, USA). RT-qPCR was performed using the Quantabio Perfecta SYBR Green Fast Mix (Cat. No. 95072-012, USA) on a Roche Light Cycler 480 instrument. The reactions were set up using 2 ng of cDNA, 250 nM primers, and 1X SYBR mix and carried out at 95 °C for 30 s, followed by 40 cycles of 95 °C for 5 s and 60 °C for 30 s, followed by melting curve analysis. RT-qPCR primers were designed such that mouse-specific primers carried more than 6 bp mismatch in the human sequence and vice versa. The expression of transcripts was normalized to *Gapdh* levels. Primers used are given in Table S5.

### 4.7. RNA-Sequencing and Bioinformatic Analysis

The integrity of the extracted RNA was analyzed using the High Sensitivity RNA Analysis Kit (Agilent, Cat. No. DNF-472-0500, Santa Clara, CA, USA) on a 12-capillary fragment analyzer. cDNA synthesis and sequencing libraries with 6 bp indices were constructed from 300–500 ng of total RNA using a TruSeq Stranded mRNA Library Preparation kit (Illumina). Libraries were validated using the Standard Sensitivity NGS Fragment Analysis Kit (Agilent, Cat. No. DNF-473-0500, USA) on a fragment analyzer and quantified using the Quant-it dsDNA Assay kit (Thermo Fisher, Cat. No. Q33120). Samples were diluted to equimolar concentrations (2 nM), pooled, and denatured. The final library dilution of 1.3 pM was subjected to paired-end sequencing of approximately 10–20 million reads per sample on a NextSeq500 using the Mid Output v2 kit (Illumina, Cat. No. 15057940, San Diego, CA, USa).

Sequencing files in fastq format were downloaded, and files from each flow cell lane were merged, followed by quality control analysis using fastQC (v0.10.1) and MultiQC (v0.9). Reads were aligned to the mouse genome using mm10 assembly by STAR (v2.5.0a) [55]. Mapped reads were used to build count matrices and gene models using Rsamtools (v2.0.0) and GenomicFeatures (v1.32.2) for expression quantification. UCSC transcripts were downloaded from Illumina iGenomes as GTF files. Reads per million were determined using GenomicAlignments (v1.16.0). DESeq2 was used for differential gene expression analysis and read count normalization. RNA-Seq data analysis of co-cultures was performed using Sargasso, as described previously [56], where the use of mixed-species co-culture systems enabled the separation of astrocyte and neuron transcripts through bioinformatic approaches. Plots for data visualization were generated using ggplot2 (v3.3.2). RNA-Seq data have been deposited at the NIH GEO database (**GSE171590**).

Gene expression heatmaps were generated using ComplexHeatmap (v2.0.0), and Gene Ontology circle plots were generated using GOplot (v1.0.2). Hypergeometric Optimization of Motif Enrichment (HOMER, v4.10) was used to identify transcription factor motifs that are enriched across different gene sets 1 kb before or 500 bp after the transcription start site. In some cases, the resulting list of enriched motifs was filtered based on expression data from in vivo Aldh1l1-GFP astrocytes purified by FACS [14].

### 4.8. ATAC-Seq and Bioinformatic Analyses

Spheres were dissociated and 50,000 cells with >80% viability determined by the Trypan Blue Exclusion method were resuspended in differentiation media, and exposed to the panel of chemicals for 30 min. Cells were pelleted at 1000 g for 3 min and washed with PBS. Cell pellets were dissolved in resuspension buffer (10 mM Tris-HCl (pH 7.4), 10 mM NaCl, 3 mM MgCl_2_) containing 0.1% NP40 (Sigma, Cat. No. 11332473001, USA), 0.1% Tween20 (Sigma, Cat. No. 11332465001, USA), and 0.01% digitonin (Promega, Cat. No. G9441, Madison, WI, USA) for lysis, which was carried out for 3 min on ice. Following lysis, nuclei were washed with resuspension buffer containing 0.1% Tween20 and pelleted at 500 g for 10 min. All buffers contained freshly prepared protease inhibitors (Roche). To the nuclei pellet, a tagmentation reaction mix containing Tagment DNA buffer and Tn5 transposase (Illumina DNA Prep Kit, Cat. No. 20018704) was added and made up to a final reaction volume of 50 µL. Reactions were incubated at 37 °C for 30 min with shaking, followed by purification using the Qiagen Min Elute Purification kit, and eluted in 10 µL EB buffer. ATAC-Seq libraries were prepared by adding indices to the purified tagmented DNA and amplified using the NEB Next High-Fidelity 2× PCR Master Mix (NEB Cat. No. M0541S, USA). PCR amplification was monitored by RT-qPCR to prevent GC and size bias, and an appropriate number of PCR cycles (10–12) was used to stop amplification before saturation [41]. Libraries were purified using AMPure XP beads, assessed for quality on a fragment analyzer, and quantified using a Quant-it dsDNA Assay kit (Thermo Fisher, Cat. No. Q33120, USA). Samples were diluted to equimolar concentrations (2 nM), pooled, and denatured. The final library dilution of 1.3 pM was subjected to paired-end sequencing of approximately 20–40 million reads per sample on NextSeq500 using the Mid Output v2 kit (Illumina Cat. No. 15057940, USA).

Sequencing files in fastq format were downloaded, and files from each flow cell lane were merged, followed by quality control analysis using fastQC (v0.10.1) and MultiQC (v0.9). Reads were aligned to the mouse genome using mm10 assembly by bowtie2 (v2.2.6). Bedgraph files were made using samtools (v1.9), and tag directories were made using the HOMER (v4.10) software suite [57]. The command findPeaks in histone mode was used to filter peaks enriched over the control sample. Enriched peaks were annotated with HOMER annotatePeaks with mm10 assembly. To identify significantly enriched peaks, getDifferentialPeaksReplicates was used with a false discovery rate of <0.01. Integrated Genome Browser-compatible files were constructed using samtools (v1.7) sort and index and deepTools (v3.1.3) bamCompare [58,59]. ATAC-Seq peaks were visualized using plotHeatmap from deepTools. ATAC-Seq data have been deposited at the NIH GEO database (**GSE171594**).

### 4.9. Chemical Reagents

Neurotransmitters and neuromodulators used were glutamate (Sigma, Cat. No. G1251, USA), GABA (Sigma, Cat. No. A2129, USA), acetylcholine (Sigma, Cat. No. A6625, USA), noradrenaline (Fisher, Cat. No. AAL0808703, USA), and serotonin (Fisher, Cat. No. AAB2126303, USA). Stock solutions (20 mM) were made using water or 0.02% DMSO. For RNA-Seq and ATAC-Seq studies, the following final concentrations of neuroactive compounds were used: Glu (500 µM), GABA (500 µM), ACh (100 µM), NAdr (100 µM), and 5HT (100 µM). For calcium imaging experiments, the following final concentrations of neuroactive compounds were used: Glu (100 µM), GABA (300 µM), ACh (20 µM), NAdr (50 µM), and 5HT (50 µM).

### 4.10. Calcium Imaging

Viral vectors for the delivery of Gfap-GCaMP6 (Addgene, Cat. No. 52124, Watertown, MA, USA) into mouse brain were made at the Viral Core Facility at the Baylor College of Medicine. Animals were deeply anesthetized with isoflurane, and after decapitation, the brain was quickly excised from the skull and submerged in an ice-cold cutting solution of 130 mM NaCl, 24 mM NaHCO_3_, 1.25 mM NaH_2_PO_4_, 3.5 mM KCl, 1.5 mM CaCl_2_, 1.5 mM MgCl_2_, and 10 mM D(+)-glucose at pH 7.4. The whole solution was gassed with 95% CO_2_–5% O_2_. Sagittal slices (300 μm) were cut using a vibratome (DSK Linear Slicer, Kyoto, Japan) with a blade (DORCO, Seoul, Korea) and transferred to extracellular ACSF solution (130 mM NaCl, 24 mM NaHCO_3_, 1.25 mM NaH_2_PO_4_, 3.5 mM KCl, 1.5 mM CaCl_2_, 1.5 mM MgCl_2_, and 10 mM D(+)-glucose at pH 7.4). Slices were incubated at room temperature for at least 1 hour prior to imaging. Slices were transferred to a recording chamber that was continuously perfused with ACSF solution at a flow rate of 2 mL/min. Slices were treated with TTX (0.5 μM) for 300 s prior to recording, and neuroactive chemicals were added after 100 s. Therefore, TTX treatment was done for at least 5 min before application of chemical, and it is unlikely that this concentration and duration of TTX would generate an action potential in neurons. Neuroactive-chemical-induced calcium responses were measured in astrocytes of the olfactory bulb from the soma and main branches using the ROI detection function in the GECIquant program and calculated by the integrated area under curve (AUC) using Prism9 software.

### 4.11. Statistical Analysis

Sample sizes and statistical tests are provided in figure legends. Significant differences are denoted by asterisks in associated graphs, and significance was assessed by one-way ANOVA or the Wilcoxon signed-rank test. Data were formally tested for normality using the Kolmogorov–Smirnov and Shapiro–Wilk tests and for the homogeneity of variance using the Levene test. When the data did not meet criteria for normality, the Wilcoxon signed-rank test was performed. Boxplots with significance were generated using ggsignif (v0.6.0) with ggplot2 (v3.3.2).

## Data Availability

The RNA-Seq and ATAC-Seq datasets generated are available at the NCBI GEO website (**GEO: GSE171595**).

## Supplementary Materials

The following are available online at https://www.mdpi.com/article/10.3390/ijms22083975/s1: Figures S1–S6 and Tables S1–S5. Figure S1: Generation of mES_astrocytes from 3D organoid-like spheres, Figure S2: Neurons trigger expression of distinct receptor subtypes in astrocytes and astrocyte development, Figure S3: Mixed-species co-culture system enables identification of ‘intrinsic’ and ‘extrinsic’ programs of astrocyte development, Figure S4: Neuronal chemical cue specific gene regulation in mES_astrocytes, Figure S5. Unique chromatin accessibility signatures are exhibited by mES_astrocytes in response to different neuroactive chemicals, Figure S6. NAdr induces enhanced calcium signaling in vivo compared to Glu, GABA, ACh and 5HT.
