# Supplementary material for: Mapping Astrocyte Transcriptional Signatures in Response to Neuroactive Compounds
Source: Int J Mol Sci. 2021 Apr 12;22(8):3975. doi: 10.3390/ijms22083975 (PMC8069033; doi:10.3390/ijms22083975)
Supplement: Supplementary file 1 [file ijms-22-03975-s001.zip › Figures S1-S6.pdf]

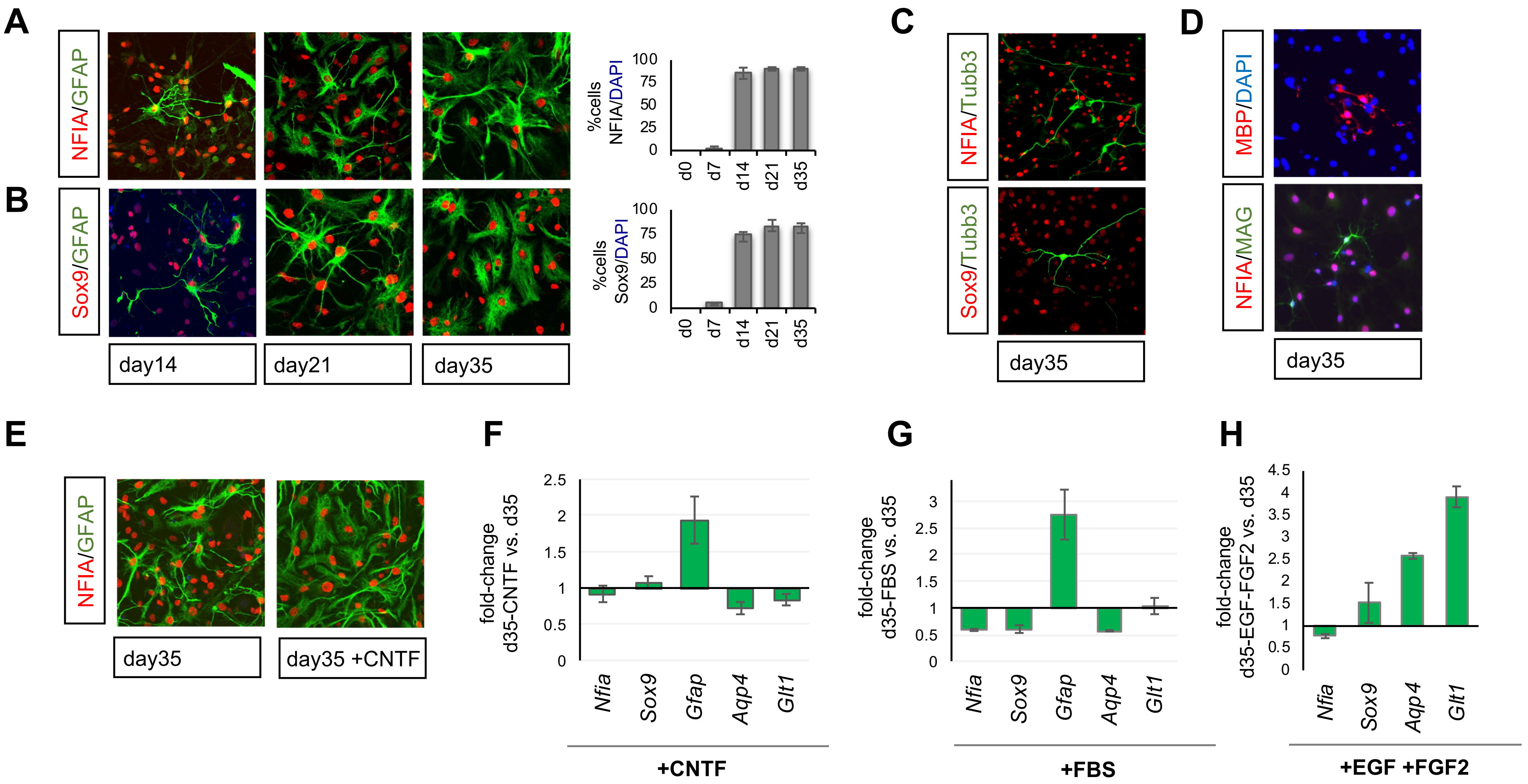

Figure S1. Generation of mES_astrocytes from 3D-organoid like spheres. (**A-B**) Analysis of NFIA (**A**) and Sox9 (**B**) in mES_astrocyte culture from day7-day35 (n=3 images each time-point) (**C**) Co-labeling of NFIA and Sox9 with neuronal marker Tubb3. (**D**) Detection of oligodendrocytes in mES_astrocytes and absence of co-labeling with NFIA. (**E**) Upregulation of GFAP in mES_astrocytes treated with CNTF (10 ng/ml) (**F-H**) RT-qPCR of astrocyte markers in day21 mES_astrocytes treated with (**F**) CNTF (10 ng/ml, (**G**) 1% FBS and (**H**) EGF+FGF2 (10 ng/ml each) till day35 (n=3). Data shown as mean ± S.D. Primer sequences are given in Supporting Table S5.

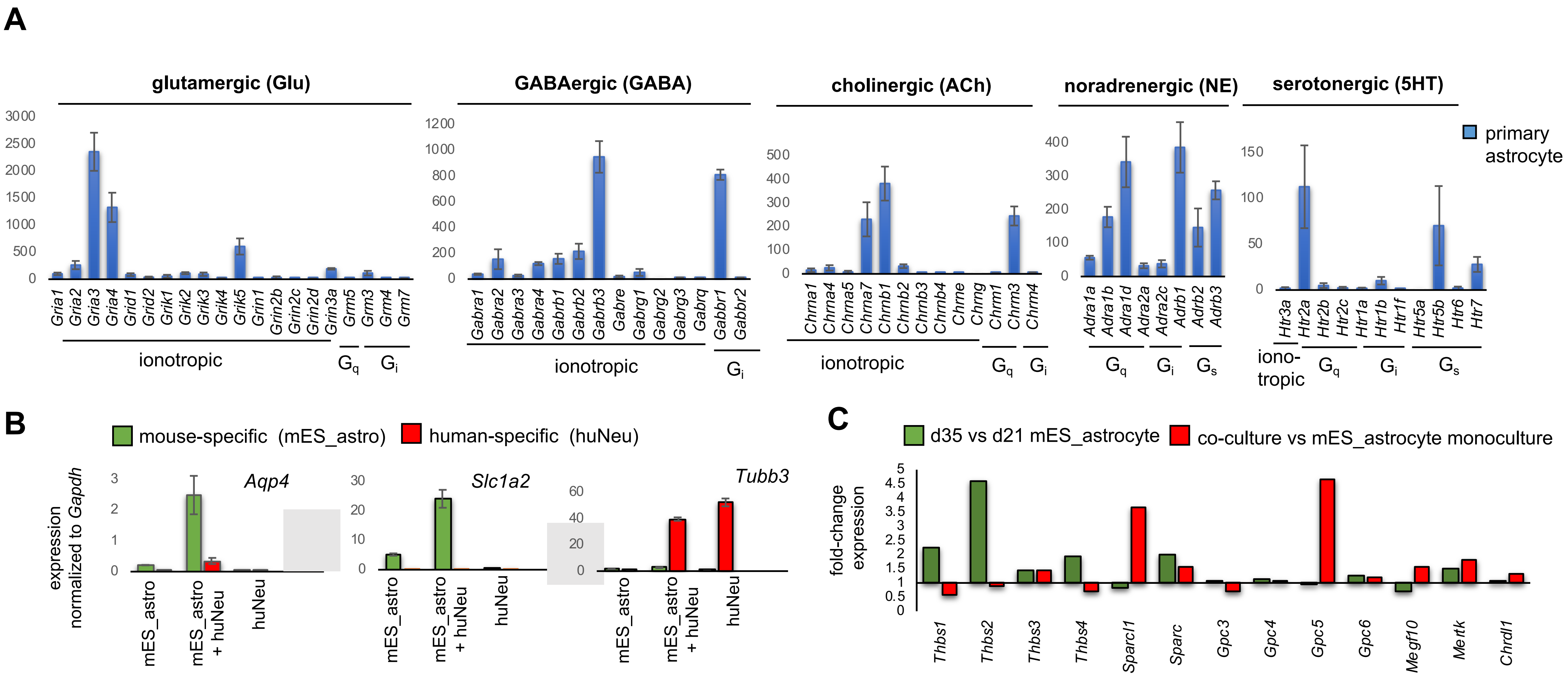

Figure S2. Neurons trigger expression of distinct receptor subtypes in astrocytes and astrocyte development. (**A**) Transcript expression levels of different receptor subtypes from RNA-Seq data of 1AS. Data shown as mean ± S.D. (**B**) RT-qPCR of mature astrocyte markers mES_astrocytes co-cultures in comparison to astrocyte or neuron monocultures. Data shown as mean ± S.D. Primer sequences are given in Supporting Table S5. (**C**) Average fold-change in transcript expression of astrocytic synapse-associated genes.

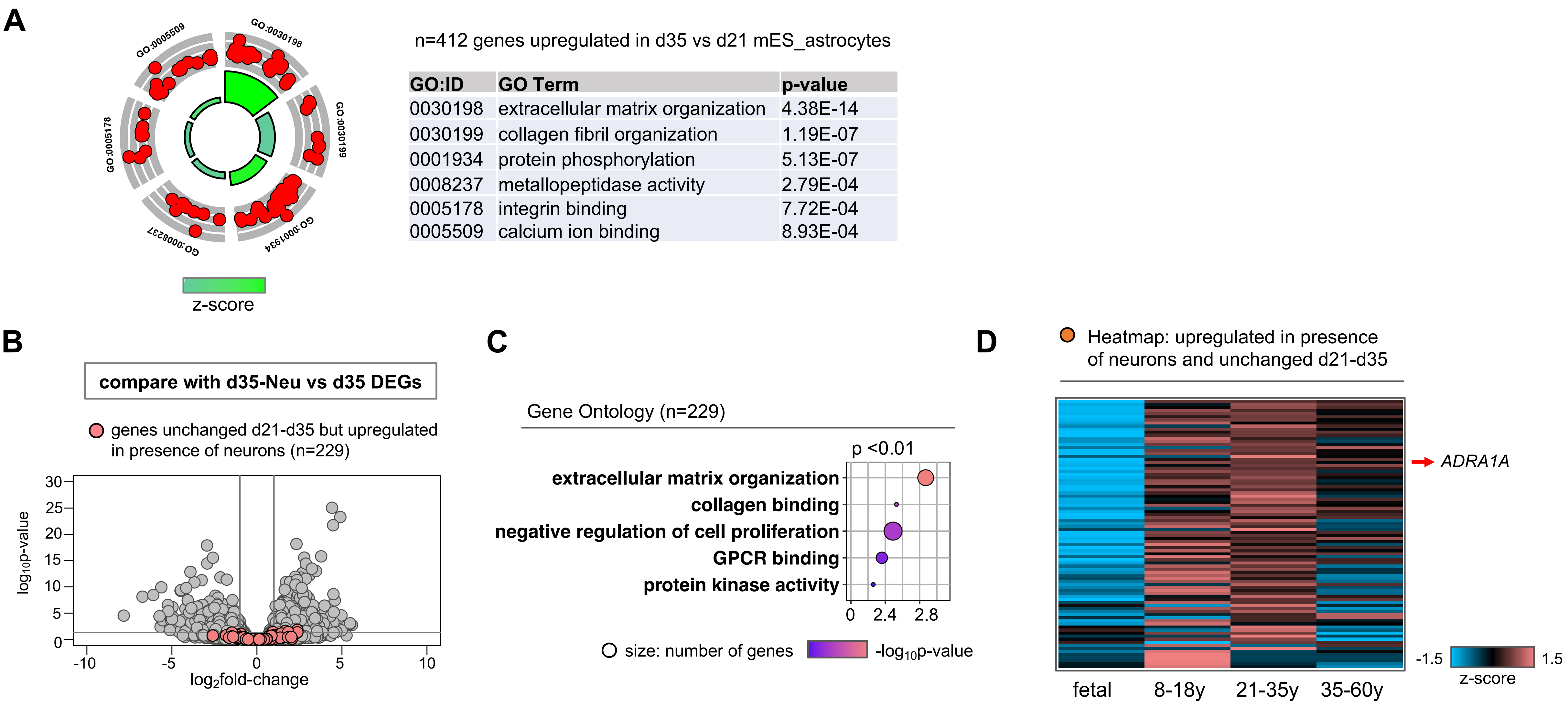

GO:ID	GO Term	p-value
0030198	extracellular matrix organization	4.38E-14
0030199	collagen fibril organization	1.19E-07
0001934	protein phosphorylation	5.13E-07
0008237	metallopeptidase activity	2.79E-04
0005178	integrin binding	7.72E-04
0005509	calcium ion binding	8.93E-04

Figure S3. Mixed-species co-culture system enables identification of 'intrinsic' and 'extrinsic' programs of astrocyte development. (**A**) GO circle plot and table showing top GO terms found in upregulated DEGs in d35 mES_astrocytes in comparison to day21 mES_astrocytes ($p<0.01$). (**B**) Comparison of DEGs shown in Figure 3B with genes differentially expressed in co-cultures to identify 229 extrinsic genes that are not differentially changed in day21-23 mES_astrocytes. (**C**) GO terms of top genes in 229 extrinsic gene set ($p <0.01$). (**D**) Heatmap showing expression levels of 229 'extrinsic' genes in human astrocytes at different ages of foetal to 60 years.

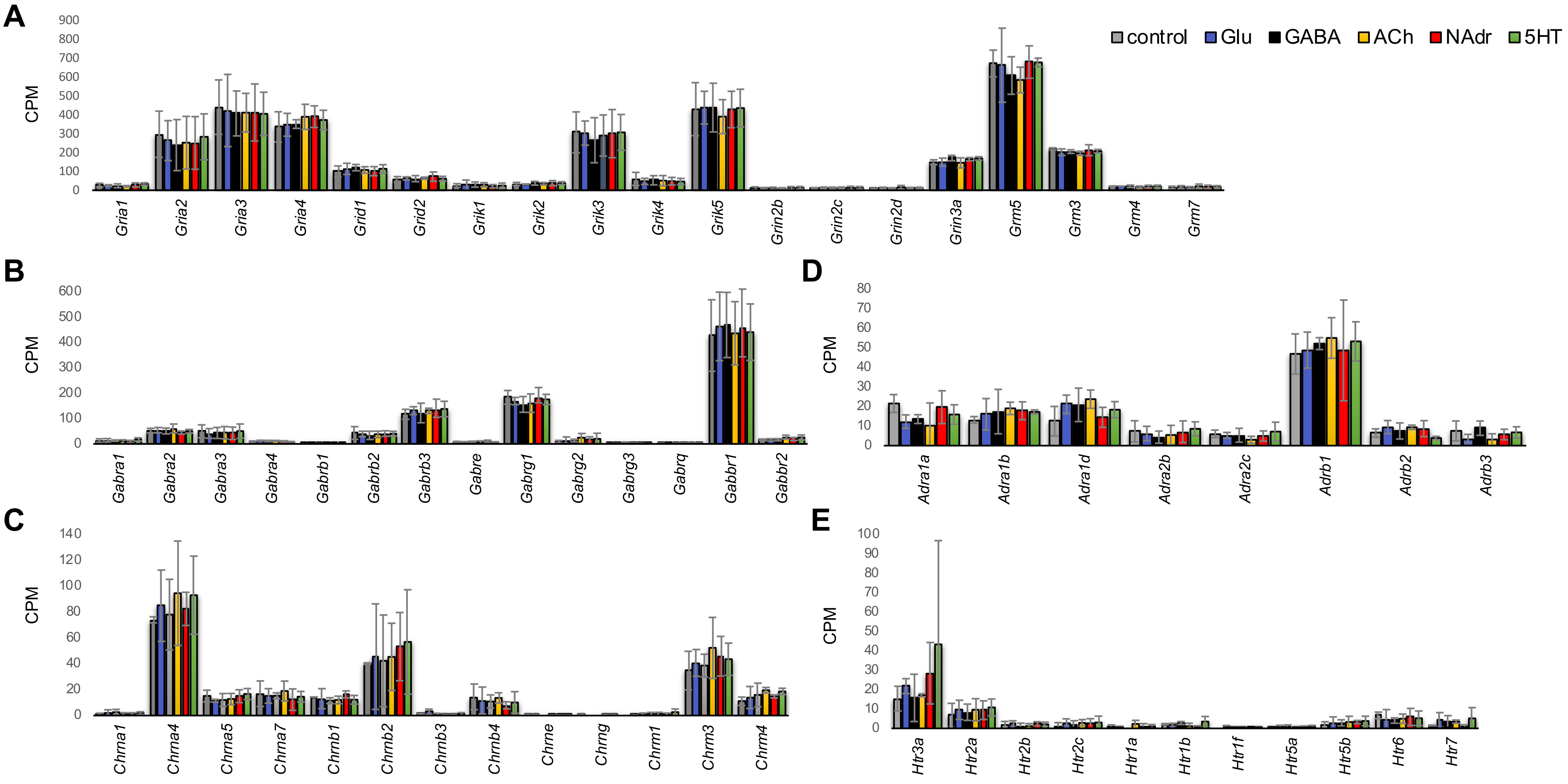

Figure S4. Neuronal chemical cue specific gene regulation in mES_astrocytes. (**A-E**) Transcript expression from RNA-Seq data of receptor subtypes in mES_astrocytes for (**A**) Glu, (**B**) GABA, (**C**) ACh, (**D**) NAdr and (**E**) 5HT receptors after exposure of mES_astrocytes to these chemicals for 30 minutes (n=3). Data shown as mean ± S.D.

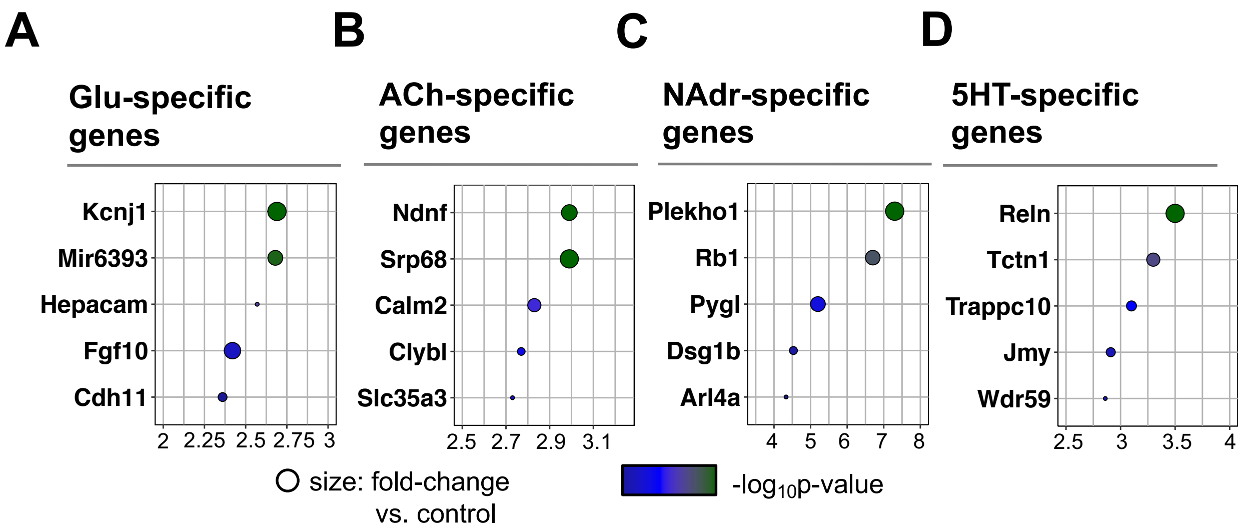

Figure S5. Unique chromatin accessibility signatures are exhibited by mES_astrocytes in response to different neuroactive chemicals. Genes with open chromatin signatures that are induced unique to (**A**) Glu, (**B**) ACh, (**C**) NAdr and (**D**) 5HT in comparison to control (n=3, $p < 0.05$).

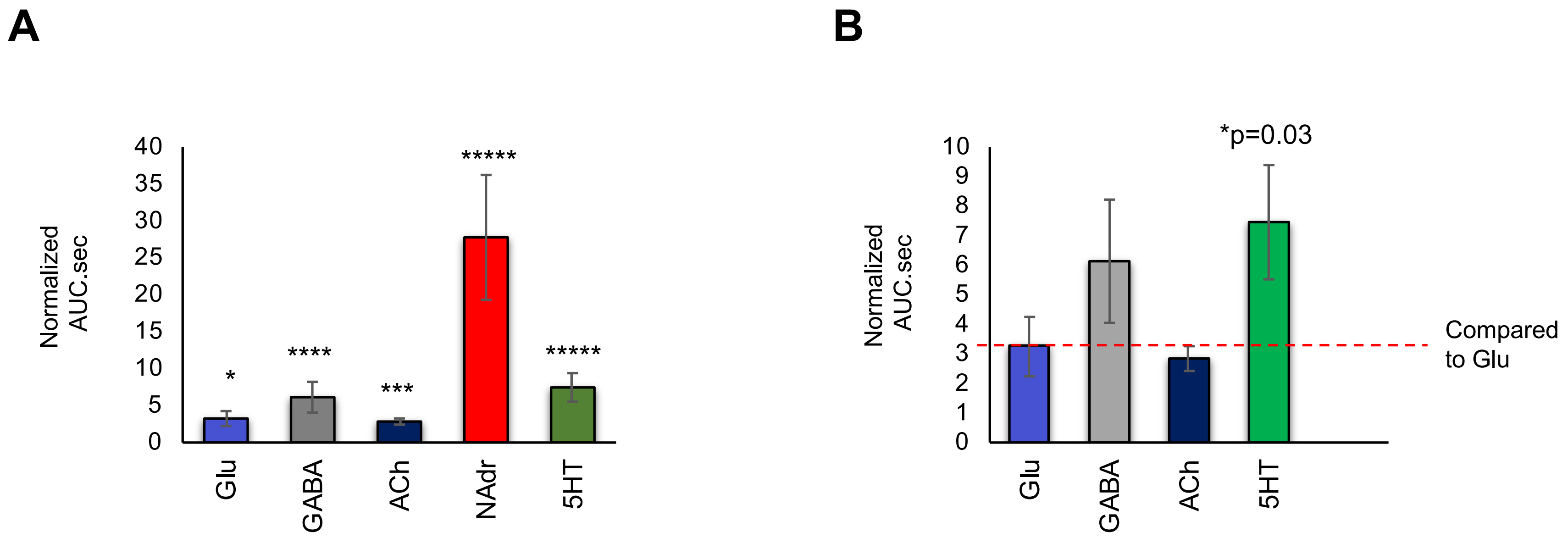

Figure S6. NAdr induces enhanced calcium signaling *in vivo* compared to Glu, GABA, ACh and 5HT. (**A**) Normalized area under curve dF/F from two-photon calcium imaging shown in Figure 6D (n=4-5, 11-12 cells each sample, *p <0.05, **p <0.01, ***p <0.001, ****p <0.0001). (**B**) Comparison of normalized calcium response of GABA, ACh, NAdr and 5HT to Glu.
